# Rehabilitation management of the chronic pain-hypertension synergy: Proposal of an evidence-informed framework

**DOI:** 10.1016/j.bjpt.2025.101229

**Published:** 2025-06-03

**Authors:** Renzo Mendoza, Craig Hensley, Jennifer Ryan, Todd Davenport

**Affiliations:** a Department of Therapy Services, University of Illinois Health Systems, 711 W. Maxwell St. Chicago, IL 60607 USA; b Department of Physical Therapy and Human Movement Sciences, Northwestern University, 645 N. Michigan Ave., Suite 1100 Chicago, IL 60611 USA; c School of Health Sciences, University of the Pacific, Stockton, California 3601 Pacific Ave., Stockton, CA 95211 USA

**Keywords:** Cardiovascular disease, Epidemiology, Examination, Exercise, Physical therapy, Risk factors

## Abstract

**Background:**

Chronic pain and hypertension (HTN) have a significant societal impact worldwide. Both lead to consumption of substantial healthcare resources and dollars. The pathophysiology of chronic pain and HTN are each complex and multifactorial. Chronic pain has been associated with an increased risk of comorbid HTN. There are numerous common factors related to the development, progression, and prognosis of patients with chronic pain and HTN. Though the cardiovascular and pain regulatory systems are structurally and functionally intertwined, the exact physiological interactions between chronic pain and HTN are less understood. Many systems, including the central and autonomic nervous systems, play a role in the development and interaction of these two complex conditions. In collaboration with other healthcare providers, physical therapists play a critical role in managing those with chronic pain and HTN.

**Objectives:**

This masterclass aims to develop physical therapists’ knowledge and understanding regarding 1) the epidemiological and physiological relationship between chronic pain and HTN, and 2) screening, examination, and management of the patient with chronic pain and HTN, including appropriate referral.

**Discussion:**

Improving understanding of the relationship between chronic pain and HTN should assist clinicians in appropriate management strategies for such patients. Physical therapy should be an essential component in the management of patients with chronic pain and HTN. By collaboratively addressing both health conditions, there is a window of opportunity to play a role in mitigating the burden of these conditions and promoting a healthier society.

## Highlights

• Chronic pain and hypertension (HTN) are intertwined global health challenges, with substantial societal and economic burdens.
• Evidence suggests a bidirectional relationship between chronic pain and HTN, influenced by shared risk factors such as metabolic syndrome, obesity, poor sleep quality, and mental health conditions.
• Physical therapists (PTs) can play a pivotal role in managing patients with chronic pain and HTN through evidence-informed screening, assessment, and interventions.
• The proposed framework integrates biopsychosocial models for personalized pain management and emphasizes blood pressure monitoring for safe, effective interventions.

## Introduction

Chronic pain and hypertension (HTN) are worldwide epidemics that can be challenging to manage. Chronic pain affects 20 % of people, while HTN affects nearly one-third of the global adult population.^1^^,^^2^ Chronic pain has been associated with multiple physical and mental health conditions, lost productivity**,** higher suicide rate**,** and substance abuse.^3^, ^4^, ^5^, ^6^, ^7^ HTN prevalence has increased across the world since its reclassification. The updated American Heart Association and American College of Cardiology defines HTN as a systolic blood pressure (BP) ≥130mmHg or a diastolic BP ≥ 80mm Hg*.* Though the exact etiology of elevated blood pressure and HTN is often not known, the effectiveness of risk reduction through lifestyle and dietary changes make it the most common preventable non-communicable disease that leads to cardiovascular disease and all-cause mortality.^3^, ^4^, ^5^, ^6^, ^7^ The total costs of chronic pain and HTN are substantial across the globe. Healthcare expenditures in the United States alone range from $261-300 billion and $131 billion for chronic pain and HTN, respectively.^8^^,^^9^

Physical therapists can play a pivotal role in managing patients with chronic pain and HTN. In this master class, we discuss the current literature regarding the relationship between chronic pain and HTN at the epidemiological and pathophysiological levels. In addition, we provide evidence suggesting that the two health conditions together comprise a syndemic.^10^ Further, we propose evidence-based guidelines regarding the screening, examination, and non-pharmacological management of patients with comorbid chronic pain and HTN.

## The relationship between chronic pain and hypertension at the population level

**Fig. 1:**
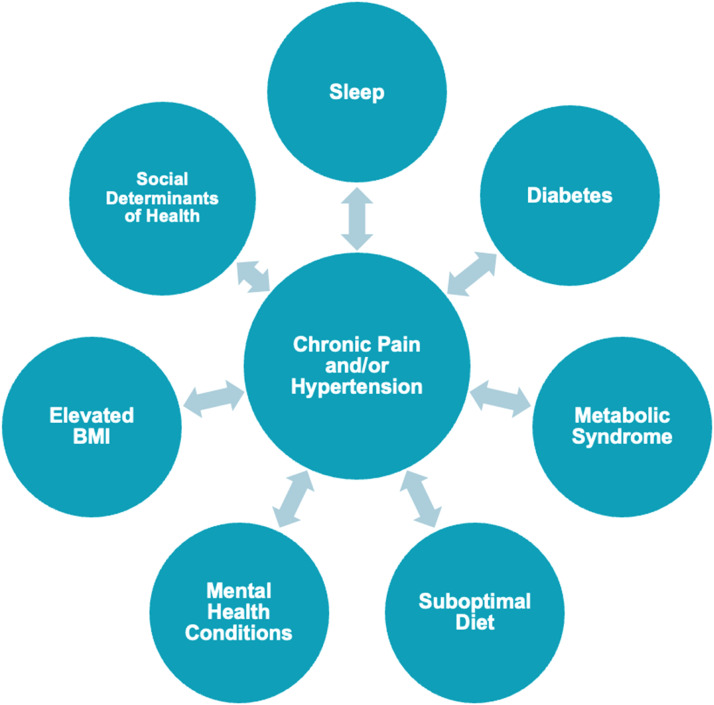
The relationship between chronic pain, hypertension, and associated factors.

Globally, up to 4 in 10 people with chronic pain have HTN.^11^ Relationships have been found between HTN and osteoarthritis, chronic low back pain (LBP), rheumatoid arthritis (RA), fibromyalgia, and complex regional pain syndrome.^2^^,^^11^ Chronic pain also has a bidirectional relationship with several hypertensive risk factors, including metabolic syndrome, suboptimal diet, poor sleep quality, elevated body mass index (BMI), abdominal obesity, Type 2 diabetes, cigarette smoking, social determinants of health, and mental health conditions.^2^^,^^12^^,^^13^ The relationship between chronic pain, HTN, and associated factors is illustrated in Fig. 1.

Individuals with chronic pain and cardiovascular disease report greater pain intensity and disability compared to those suffering from chronic pain alone.^2^^,^^14^ Moreover, a recent cross-sectional study has shown that the presence of comorbidities is associated with higher odds of daily pain, pain impact, and pain intensity.^15^ Individuals with chronic pain and comorbidities are less likely to receive appropriate care, have a poorer prognosis, and account for higher payment cost.^16^^,^^17^ Further, many of the associated factors listed in Fig. 1 have been shown to influence individuals’ pain intensity, symptom distribution, and pain reactivity.^2^^,^^15^

The interplay between chronic pain and HTN can be considered a syndemic. This term refers to the coexistence and interaction of multiple health conditions and factors that synergistically worsen prognosis and outcomes, creating a compounded health burden greater than the sum of its parts.^10^ Understanding this relationship allows physical therapists to develop more holistic and effective interventions that address these intertwined factors, ultimately leading to better patient outcomes.

## Potential mechanisms mediating the relationship between blood pressure and pain

### Hemodynamic response to noxious stimuli in healthy individuals

The cardiovascular and pain regulatory systems are structurally and functionally intertwined. In healthy individuals, these interconnections produce blood pressure (BP)-related hypoalgesia, in which a noxious stimulus triggers elevated BP to initiate a reduction in pain experience (Supplemental material 1).^18^^,^^19^ A noxious stimulus travels through the somato-autonomic reflex, which increases BP through sympathetic arousal (Fig. 2a). Increased BP leads to baroreceptor stimulation, triggering the baroreflex and associated pathway in the central nervous system (CNS). The baroreceptor system is vital to cardiovascular regulation. It consists of a negative feedback loop in which electrical activity changes in arterial baroreceptors, resulting from BP alteration, initiate compensatory changes in heart rate, cardiac contractility, and vascular tone.^20^ This feedback system processes information within the cardiovascular cranial nerve branches into the lower brain stem. Brain signaling reinforces sympathetic output and descending modulation of noxious stimuli via noradrenergic pathways to reduce the painful experience. Once reduction in noxious and pain signaling occurs, there is a reduction in activation within this baroreflex and associated pathways, allowing BP to return to baseline.

**Fig. 2a:**
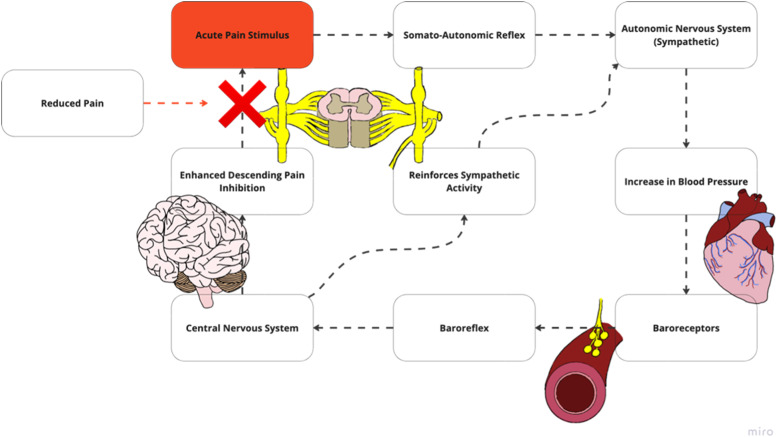
(A) Along with the secondary projections that sends signals to supraspinal centers, the noxious stimulus additionally travels through the somato-autonomic reflex, which increases sympathetic arousal and produces increased blood pressure. (B) Increased blood pressure leads to baroreceptor stimulation which triggers the baroreflex, inputting sensory information into the central nervous system. Alterations in baroreceptor sensitivity may limit the ability for the autonomic system to stimulate the central autonomic network which includes the nucleus of the solitary tract and the locus coeruleus. (C) Signals from the baroreceptor triggers the central autonomic network. (D) This reinforces sympathetic output and pain inhibition via noradrenergic pathways, decreasing pain stimulus. Adapted with permission from Bruehl et al. pain and blood pressure model^26^.

Although BP-related hypoalgesia has been postulated to respond to acute pain stimulus, it is important to highlight this mechanism has demonstrated mixed results in conjunction with exercise. Exercise inherently is thought to produce an increase in BP that attenuates the baroreceptor related mechanism.^21^ Research on isometric exercises found that increases in BP has not consistently corresponded to alterations in pain perception.^22^ These findings suggest that other physiological mechanisms may be at play aside BP alone such as endogenous opioids, cannabinoids and growth hormones which have been hypothesized to be produced during exercise.^21^

### Alterations in hemodynamic response to noxious stimuli in individuals with chronic pain

Altered central nociceptive processing may occur with chronic pain, leading to nociplastic pain.^23^ While various mechanisms are associated with nociplastic pain, including hyperexcitability of nociceptive pathways, another potential feature of the condition is impaired descending inhibitory mechanisms. Descending noradrenergic inhibition can be profoundly effective in modulating pain, and may be impaired in individuals with HTN and chronic pain.^24^^,^^25^ Chronic pain is associated with reduced magnitude of BP-related hypoalgesia, suggesting maladaptive processes occurring within the cardiovascular regulatory systems (Supplemental Material 1). These are potentially relevant to comorbid cardiovascular risk in chronic pain.^26^ Possible reasons for this altered relationship between BP and pain sensitivity include exhaustion of certain pain-inhibiting pathways, changes in baroreceptor function, increased inflammation, and other contributing factors (Fig. 2b).

**Fig. 2b:**
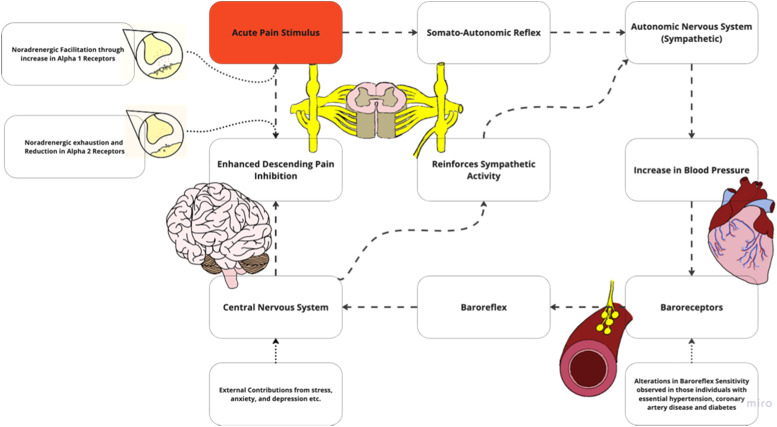
Alterations in the pain and blood pressure model may include noradrenergic exhaustion where a reduction in norepinephrine which normally modulates pain. Another possibility in this region includes facilitatory mechanisms with changes in receptor type may promote symptoms. Adapted with permission from Bruehl et al. alterations in the pain and blood pressure model^26^.

#### Noradrenergic exhaustion

In chronic pain, persistent and excessive antinociceptive or endogenous pain blocking demands may eventually exhaust noradrenergic pathways^24^, ^25^, ^26^ (Fig. 2b). It additionally has been hypothesized that persistent nociceptive input and depletion of norepinephrine reserve leads to a reduction of receptor types within the dorsal horn. These two factors lead to an inability of norepinephrine to stimulate pain inhibition at the spinal level. Thus, pain is left uninhibited, allowing for a continuous cycle to increase BP. A reduction in noradrenergic receptors has also been shown to contribute to HTN by affecting renal function.^27^ When the renin angiotensin aldosterone system is overly active, it can cause persistent vasoconstriction, increase blood volume, overstimulate the sympathetic nervous system, and cause vascular remodeling.^28^^,^^29^ This can lead to many systemic conditions, including essential HTN, renovascular HTN, heart failure, and chronic kidney disease.^29^

#### Noradrenergic facilitation

Alterations in noradrenergic facilitatory mechanisms result in noradrenaline producing more nociceptive drive rather than inhibiting (Fig. 2b). This stems in part from descending facilitatory mechanisms and/or promotion of pain input.^30^^,^^31^ Persistent pain has been shown to increase excitability and sensitivity of spinal neurons, further driving nociplastic changes.^32^, ^33^, ^34^, ^35^ These CNS changes also include alterations in receptor type which have been hypothesized to increase firing when bound by norepinephrine. Noradrenergic facilitation increases spinal cord excitability when exposed to an influx of norepinephrine. Pain is further facilitated, with continuous driving of BP elevation.

#### Baroreceptor habituation

It is important to highlight that baroreceptor efficiency is often determined through the measurement of baroreceptor sensitivity.^36^ This measurement is defined as the change in the R-R interval in milliseconds per mmHg change in systolic BP and is the most established parameter describing the function of the baroreflex loop.^36^ Reductions in baroreceptor sensitivity have been demonstrated in those with HTN^36^ and chronic pain states, including LBP, RA, fibromyalgia, temporomandibular disorders, and irritable bowel syndrome.^26^^,^^37^ Reduced sensitivity has also been observed in the presence of several health conditions and other factors such as anxiety, diabetes, chronic smoking, and obesity.^36^^,^^38^ Alterations in pain regulatory processes associated with chronic pain can be associated with changes in baroreceptor sensitivity.^36^ Changes in baroreceptor sensitivity may occur due to an altered baroreceptor firing threshold. Thus, with changes in baroreceptor activation, an inability to reach the central nervous system occurs and ultimately influences pain inhibition (Fig. 2b).

#### Systemic inflammation

A bi-directional relationship is possible between systemic inflammation and chronic pain and/or HTN. Elevated levels of systemic inflammation have been associated with higher odds of having pain and contributes to initiation, progression, and maintenance of HTN.^39^, ^40^, ^41^, ^42^, ^43^ Chronic pain has been shown to contribute to a persistent inflammatory state at both the peripheral and central level. This results in neuroplastic changes, including peripheral and central sensitization.^44^ HTN has also been proposed to trigger low grade systemic and vascular inflammation, leading to oxidative stress, and alterations in the extracellular matrix composition**.**^45^^,^^46^

#### Other contributory factors

Stress, anxiety, sleep deprivation, and depression have been suggested to play a role in this mechanistic model linking chronic pain and HTN (Supplementary Material 2).^18^ These factors all relate to chronic stress and psychological distress which, both in animal and human studies, are related to CNS dysregulation and a neuroinflammatory state linked to chronic pain syndromes and HTN.^37^^,^^47^ This relationship underscores the importance of a comprehensive biopsychosocial screen, a practice that has long been an integral piece of the physical therapy examination.^23^^,^^48^^,^^49^

## Patient assessment

### Evaluate for health conditions requiring referral

Chronic pain conditions may mask sinister pathology requiring assessment and intervention from another health care practitioner. Unfortunately, clinicians are more likely to discount symptoms that patients with chronic pain report and are less inclined to provide intervention, particularly when evidence for a particular pathology is lacking and/or the patient reports high pain intensity.^50^^,^^51^ It is imperative that health care providers continue to screen and examine throughout the episode of care, even if patients are improving. For instance, Hensley and Emerson report on a patient with chronic left upper quarter pain who was improving with management provided by the physical therapist. A change in presentation during the episode of care warranted referral for further medical workup, leading to a diagnosis of lung cancer.^52^ Moreover, patients with chronic pain and HTN should be evaluated for signs and symptoms consistent with post-exertional malaise/post-exertional neuroimmune exhaustion (PEM/PENE),^53^ because pain is a common component of PEM/PENE. PEM/PENE is a clinical hallmark of myalgic encephalomyelitis (ME) and ME-like conditions, such as subtypes of Long Covid^54^, ^55^, ^56^, ^57^ and post-treatment Lyme disease.^58^ These conditions are important to be aware of because exercise recovery responses are impaired in people with PEM/PENE.^59^, ^60^, ^61^, ^62^, ^63^ In those with PEM/PENE, exercise should not be considered as a frontline intervention.^64^

### Identify predominant pain mechanism(s) involved

A mechanism-based approach to pain has been proposed to improve pain management. A mechanisms-based approach integrates and builds on the biopsychosocial models by defining specific pathobiology in pain processing, and external factors influence an individuals’ pain experience.^23^ Pathobiological categories in pain processing include nociceptive, nociplastic, and neuropathic pain. These are further defined in Table 1. Recent recommendations have proposed features that discriminate between these three categories.^65^^,^^66^ It is important to recognize that more than one category can simultaneously contribute to an individual’s pain experience. In addition, consideration of pain-relevant psychological and motor factors must be given, as they may play a role in the maintenance of an individual's pain.^23^ Interventions have been proposed that can be matched to specific pain mechanisms.^23^^,^^67^ For instance, a patient with LBP predominantly driven by a nociceptive source can be managed with interventions that target biomechanics, such as posture education, strengthening weak musculature, and stretching structures deemed inflexible/hypomobile. Alternatively, if the pain is driven predominantly from a nociplastic source, it is unlikely that treatment targeting mechanics alone will improve outcomes. Pain neuroscience education (PNE) can be a crucial addition to exercise and manual therapy to assist these patients.^68^^,^^69^ It should be noted that there is a paucity of evidence demonstrating that a mechanism-based approach to pain provides better benefits to patient outcomes compared to a thorough biopsychosocial assessment and person-centered management plan.

**Table 1:** Identifying and differentiating between pain mechanisms^65^^,^^70^.

	Nociceptive	Nociplastic	Neuropathic
Pain location	Local	Diffuse, widespread, multisite, and/or poorly localized. Varying distribution over time	Distributed to nerve injury/ pathology
Mechanism of injury	Clear, proportionate	Less clear or unclear, disproportionate	Consistent with nerve injury/ pathology
Pain behavior	Proportionate to injury and healing stage	Higher irritability Prolonged healing time	Consistent with nerve injury/ pathology
Pain descriptors	Sharp in acute stage Dull ache, throb at rest	Combination of nociceptive/ neuropathic descriptors	Lancinating, shooting, electric-like, burning, hot
Other subjective findings	+ response to anti-inflammatory medication	Fatigue, sleep, cognitive, mood, and/or gastrointestinal disturbance(s) Hypersensitivity to non-musculoskeletal stimuli (e.g., sound, heat, cold)	Paresthesia
Example screening tools		Central Sensitization Inventory score above^70^	1. painDETECT^71^, ^72^, ^73^ 2. Leads Assessment of Neuropathic Signs and Symptoms^74^
Movement testing	1. Pain reported in particular phases of movement 2. Consistent with a specific health condition	1. Pain reported through multiple phases of movement with multiple movement problems 2. Less consistent or inconsistent with a specific health condition(s)	Consistent with nerve injury/ pathology
Other objective findings	1. Warm, swelling, skin color changes indicative of local inflammation 2. Positive special tests linked to body structure(s)	1. Hyperalgesia and/or allodynia 2. Pain pressure thresholds- lower at distant site, contralateral extremity, or throughout upper/lower quarter) 3. Conditioned pain modulation- reduced or no change 4. Temporal summation- increased	Positive central or peripheral neural exam findings 1. Muscle performance 2. Sensation 3. Deep tendon reflexes 4. Upper motor neuron testing 5. Neurodynamics 6. Other neural testing (i.e. tapping over nerve reproduces symptoms)

**Nociceptive pain**- pain arising from activation of nociceptors in non-neural tissues, which is traditionally associated with acute injury, inflammation, or mechanical irritant.

**Nociplastic pain-** pain that stems from alterations of nociceptive processing within the central nervous system and often there is no clear evidence of tissue damage nor somatosensory disease.

**Neuropathic pain** symptoms associated with a specific lesion or disease within the somatosensory system.

While the standard patient interview and physical examination is critical, physical therapists may use additional testing, including quantitative sensory testing (QST), to help determine pain mechanism(s) involved. These tests include palpation, pain pressure threshold, temporal summation, and conditioned pain modulation. It should be noted that these tests have several limitations as stand-alone pieces of an evaluation.^12^^,^^75^ Firstly, they are indirect measures of CNS excitability and inhibition of pain pathways. Secondly, there is a general lack of consensus on normative and predictive values. Thirdly, findings from these tests do not always correlate with symptoms intensity or improvement in symptoms.^76^, ^77^, ^78^ Finally, funding for equipment and time to perform QST may not be widely available across rehabilitation settings. There are, however, cheaper, alternative testing equipment that can be used to perform QST measures.^79^^,^^80^

#### Palpation

Palpating the area where pain is reported and areas remote from the site of pain can provide valuable clues. With palpation, clinicians should be looking for hyperalgesia (i.e., augmented response to noxious stimuli) and allodynia (i.e., pain evoked with a non-noxious stimulus). If either of these palpation tests are positive, particularly distant from the site of pain, a nociplastic pain mechanism should be considered.^23^^,^^80^

#### Pain pressure threshold (PPT)

PPT testing is a method used to objectively measure hyperalgesia. With the use of a pain algometer, pressure can be applied at sites local and distant to the pain location at a rate of about 30 kPa/s. Alternatively, a modified 10 ml syringe can be used as a substitute.^81^ Moderate evidence has shown that there may be a relationship between brain alteration and PPT.^82^ Further, patients are less likely to respond to physical therapy management when a reduction in PPT is found.^83^

#### Conditioned pain modulation

The clinician measures PPT at a distant site before and after inducing a noxious stimulus (BP cuff or cold-water immersion). This is a proxy measure of descending pain inhibition and exercise-induced hypoalgesia (EIH). If PPT is reduced or no change is found, a nociplastic condition is suspected.^84^

#### Temporal summation

Temporal summation is the term used to describe the phenomenon of windup. Windup is characterized by the gradual amplification activity in the dorsal horn cells following repetitive stimulation of primary afferent C-fibers.^81^ A repetitive noxious stimulus with a 0.7 mm hair/filament is applied with a frequency of 1/s for 30s in an area of hyperalgesia. Patients are asked to rate the pain intensity induced by the first and last stimuli.^81^ An increase in pain intensity between the first and last stimulus indicates enhanced central excitability and reflects facilitated temporal summation. This response is considered a clinical sign of central sensitization. An alternative proxy measure could be a functional test, such as the six-minute walk test.^85^ Elevation in temporal summation suggests that the patient is less likely to respond to physical therapy intervention.^83^

### Assessment of vital signs

**Fig. 3:**
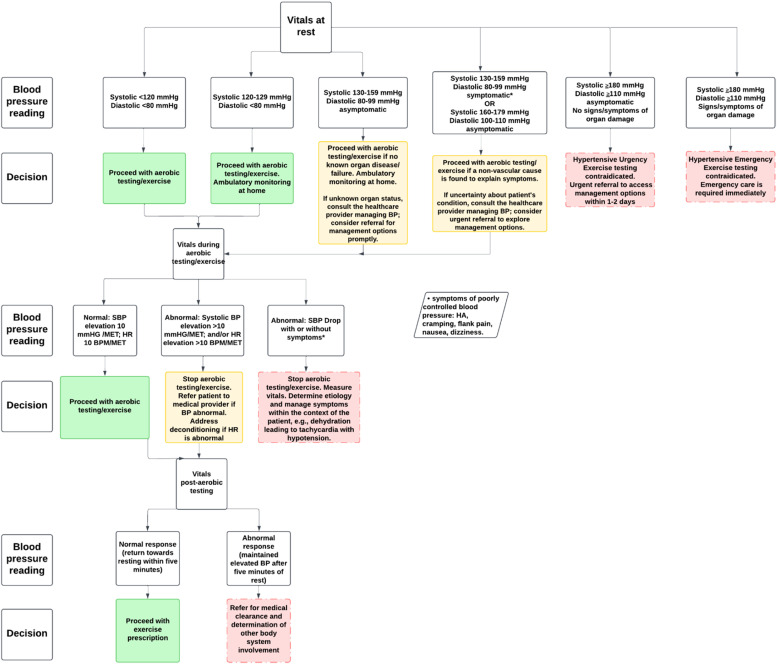
Decision-making algorithm to identify and manage patients with elevated blood pressure.

Physical therapists possess the expertise to assess vital signs throughout the episode of care, not only to conduct health screenings and gather patient history but also to ensure safety and appropriateness prior to initiating exercise interventions. They are positioned to expedite the World Health Organization’s initiative to reduce prevalence of undiagnosed or uncontrolled HTN.^1^ The specific parameters to attend to in order to mitigate threats to accurate BP measurement is available from the American Heart Association for reference.^86^ Appropriate BP management is likely to reduce the risk of negative cardiovascular/cerebrovascular-related sequelae, and improve societal health.^87^, ^88^, ^89^, ^90^ Vital sign assessment should include monitoring at rest, during aerobic activity, and during recovery. This testing approach can reveal BP readings that place the patient at risk for an adverse event.^1^^,^^12^^,^^16^^,^^91^^,^^92^ Fig. 3 provides guidelines for assessment and management based on patient vitals during different stages of rest, aerobic testing/exercise, and during recovery to ensure safety and optimal health outcomes. It includes specific BP measures and corresponding clinical decision making.

#### Resting blood pressure

At rest, normal BP (<120/80 mmHg) allows for safe exercise testing if there is no history of organ disease or failure; any uncertainty requires consultation with another healthcare provider. Any reading between >120/80 and <180/120 mmHg in a patient without HTN diagnosis and symptoms (dizziness, nausea, tinnitus, headache, or pain unrelated to their primary diagnosis) necessitates at home BP measurements **for at least 1 week**, close in clinic monitoring, and referral to an appropriate healthcare provider for additional monitoring (usually 2-3 office visits at 1-4 week intervals).^93^

In patients with known HTN, or elevated BP (120-129/<80 mmHg), at home and close in clinic monitoring is required throughout the episode of care. Readings between 130-159/80-99 mmHg in the patient with diagnosed HTN and on medication is considered to be uncontrolled. If asymptomatic, submaximal aerobic testing may proceed, education on lifestyle modification that is evidenced to reduce BP, and a referral to the healthcare practitioner managing the patients BP (general practitioner, cardiologist, etc) is required to ensure medical management is optimal. If a patient presents with symptoms such as headache, chest pain, tinnitus, or vision changes, exercise testing is contraindicated, and prompt medical evaluation is required. Any systolic reading ≥180 mmHg and/or diastolic reading ≥110 mmHg is a contraindication to resistance training and aerobic activity.^94^^,^^95^ The literature varies on the upper limit of BP readings as contraindications to exercise. However, we recommend the lowest limit as a conservative approach in individuals with chronic pain, as there are likely many other variables that impact exercise intensity in this population. Asymptomatic patients should seek care for management options within 1-2 days after detection, as this is considered a hypertensive urgency case. These patients could still benefit from other interventions prior to comprehensive testing. If the patient is symptomatic or shows signs of organ damage, this is considered a hypertensive emergency requiring access to emergency care.^96^^,^^97^

#### Blood pressure during aerobic activity

There is growing evidence for the utility of high BP responses to submaximal aerobic activity (60-70 % maximum HR intensity).^98^, ^99^, ^100^ Recent data have shown that systolic BP ≥160 mmHg during submaximal exercise testing is associated with an increased risk for a major cardiovascular event.^98^^,^^101^ During aerobic activity, normal vital responses include a gradual increase in systolic BP of approximately 10 mmHg per metabolic equivalent (MET) and a corresponding rise in heart rate (HR) of around 10 beats per minute (BPM) per MET as measured in normal adults.^102^ These increases reflect the body’s appropriate physiological adaptation to the increased demand for oxygen during physical activity. Estimated MET levels using the Fitness Registry and the Importance of Exercise National Database (FRIEND) equations can assist the clinician with accurately estimating MET level increases.^103^ For instance, walking at 2.41 kilometers per hour (km/h) at 0 % grade is equal to approximately 3 METs. Increasing speed to 3.22 km/h on a 3.5 % grade equates to approximately 4 METs. In patients taking beta blocker medication, HR response is blunted. The rate of perceived exertion (RPE) scale should be used in supplement to vital sign measurement.^104^ The RPE is also a useful tool to guide exercise prescription in cases where the patient cannot tolerate aerobic testing. Regardless, an unexplained drop in systolic BP, no increase in systolic BP, or an exaggerated elevation in BP or HR may suggest poor cardiovascular response, deconditioning, or other potential cardiovascular abnormalities. The largest study available in the literature that measured exercise testing responses in patients with cardiovascular disease offers a sense of the variance from normal responses as driven by disease.^105^ There is no database for standardized exercise testing responses for individuals with chronic pain. The clinician will need to allow self-selected intensities with ongoing vital sign monitoring due to variations in exercise intensity as driven by pain intensity.

If a patient without a history of HTN is found to have elevated BP in response to aerobic activity, careful monitoring of vitals over time is needed to confirm a diagnosis of HTN. In patients with HTN, abnormal responses require immediate cessation of aerobic activity with prompt referral for a medical evaluation to assess the underlying causes and determine if further diagnostic testing or treatment is needed. Additionally, if there are concerns about the patient’s cardiovascular health, further monitoring and a more comprehensive evaluation may be warranted. Aerobic activity should be terminated if the patient develops symptoms (dyspnea, extreme fatigue, faintness, chest pain, unsteadiness, change in mentation), signs of poor peripheral perfusion (cyanosis, cramping, or pallor), there is a drop in systolic BP > 10mmHg, or there is a BP reading that exceeds **250/115** **mmHg**.^106^ Finally, it should be noted that manual measurement of BP is necessary during exercise. Automatic oscillometric BP measurement, while useful at rest, cannot accurately measure BP during exercise.^107^

#### Blood pressure during recovery

There is also growing utility for the use of BP and HR response after aerobic activity.^87^, ^88^, ^89^, ^90^^,^^98^^,^^108^, ^109^, ^110^ For instance, in middle-aged men, if systolic BP is >195 mmHg two minutes after aerobic testing, there is a 69 % increased risk for a future myocardial infarction.^111^ In contrast, recent data have shown a 1.27 increases odds of a major cardiovascular event for every 10 mmHg increase in diastolic BP three minutes after exercise.^112^ This is understandable because an elevation in diastolic BP reduces the opportunity for the myocardial perfusion between beats. BP should be taken at completion of aerobic activity.^113^ BP should progressively trend to normal resting levels over the course of 5-6 min post-aerobic activity. HR should decrease at least 12 BPM in the first minute and return to near resting.^113^ If BP remains elevated beyond 5-6 min during recovery, it is considered an abnormal response and may signal an underlying issue, such as persistent HTN or poor cardiovascular conditioning. In such cases, the patient should undergo further medical evaluation.

### Determine contributing factors to chronic pain and HTN

Multiple common risk factors should be considered when evaluating a patient with chronic pain and HTN. Assessment tools are identified and described in Table 1. As able, the health care team should work with the patient to mitigate these risk factors. Providers should also consider pain medications that can elevate BP. Risk of systolic/diastolic HTN can increase with consumption of non-steroidal anti-inflammatory drugs (e.g., naproxen), serotonin norepinephrine reuptake inhibitors (e.g., duloxetine), and tricyclic antidepressants (e.g., amitriptyline).^93^^,^^114^

## Intervention for the patient with comorbid chronic pain and hypertension

Given the relationship described above between chronic pain and HTN, interventions could be designed to target risk factors for chronic pain and HTN and the health conditions themselves. Further, interventions targeting pain management may also reduce BP.

### Education

Educational material should be provided in an accessible format to the patient. Education should be provided regarding:

1) What their BP reading means relative to healthy BP.
2) How to accurately measure BP at home for consistent comparison.
3) Warning signs and symptoms that require a consultation with a physician or an emergency room visit.
4) The potential bi-directional relationship between chronic pain and HTN, and how management targeting a patient-centered approach can assist with management of both.
5) Associated risk factors, particularly modifiable risk factors like stress, sleep, physical activity, and diet, related to chronic pain and HTN. Stress, for example, can be addressed through teaching coping strategies using the eliminate-change-accept approach and/or by increasing greenspace exposure.^115^ Educating on the importance of sleep and sleep hygiene can be tailored to address any sleep-related dysfunction.^116^ Clinicians should consider a referral to a psychologist, sleep specialist, and/or dietitian when deemed appropriate.
6) Factors related to predominant pain mechanism(s). Modifiable biomechanical factors that may be further perpetuating symptoms should be discussed. PNE could be incorporated when the clinical picture is dominated by nociplastic pain, or when maladaptive pain perceptions and/or coping strategies are present.^68^ Clinicians should aim to improve patients’ knowledge of pain processing within the nervous system, engaging patients in a discussion regarding their pain and attempting to reconceptualize the patient’s understanding and beliefs about their pain as needed.^117^ Recent evidence has shown beneficial effects of PNE in chronic musculoskeletal pain^69^ and fibromyalgia.^117^

### Exercise

Exercise can provide both acute and chronic changes in pain and BP.^118^, ^119^, ^120^ While exercise can increase BP during activity, there is a drop in BP for up to several hours after exercise in those with and without HTN.^118^^,^^121^ As suggested above, vitals should be measured throughout the plan of care to ensure safe delivery of exercise. Chronic adaptations in BP response to various forms of exercise are outlined in Supplementary Material 3. It should be noted that current data do not differentiate chronic changes in exercise between those with and without chronic pain. These changes should be used and interpreted with caution in patients with chronic pain.

Several mechanisms have been suggested as to why exercise is effective for managing chronic pain and HTN. Namely, there are improvements in pain regulation systems through EIH; improvements in autonomic regulation through decreasing sympathetic tone and vagal inhibition; increases in circulatory and intraarticular anti-inflammatory markers, and changes in brain chemical makeup, activation, and connectivity.^122^

If deemed safe to begin an exercise program, factors such as current activity level, patient’s previous response to exercise, patient beliefs, readiness to begin an exercise program, predominant pain mechanism(s), health conditions, and current medications need to be considered. Integrating exercise in the plan of care in those with chronic pain can be challenging. Nijs et al outline a stepwise approach to assist with implementing exercise for patients with chronic pain.^123^ This includes an individually tailored plan that may involve:

1) Goal setting plus identifying body functions, activity limitations, participation restrictions, and contextual factors related to pain
2) Shared decision making
3) Tackling maladaptive beliefs about pain and physical activity
4) Improving lifestyle factors that can optimize exercise, such as sleep, diet, and stress
5) Identification of activities that are avoided (managed with graded activity) versus persisted (managed with pacing and acceptance-based interventions).

Table 2 provides proposed guidelines for clinical-decision making regarding exercise parameters in patients with chronic pain and HTN.^119^^,^^122^^,^^124^, ^125^, ^126^ Aerobic, resistance, and isometric exercise have demonstrated effectiveness in managing patients with chronic pain or HTN.^119^^,^^122^^,^^124^, ^125^, ^126^ Exercise can be tailored to best meet the demands of the patient. For instance, if a patient has significant pain at the knee yet can tolerate regional exercise at other body sites, clinicians can consider titrating exercise at these other and less painful sites.^127^

**Table 2:** Beginning clinical decision-making exercise parameter guidelines for those deemed safe to exercise with chronic pain^113^^,^^128^, ^129^, ^130^, ^131^.

Factor	Low-moderate intensity exercise	Moderate-high intensity exercise
**Current activity level**	Sedentary-low	Moderate-high
**Previous exercise response**	Negative	Positive
**Exercise/movement beliefs**	Negative	Positive
**Pain intensity/irritability**	Moderate-high	Low
**Vitals at baseline**	130-179/80-109 mmHg for asymptomatic cases of HTN	<130/80 mm Hg
**Vitals during submaximal aerobic activity**	Vitals responding inappropriately to testing per metabolic equivalent	Vitals responding appropriately to testing per metabolic equivalent
**Vitals post-aerobic activity**	Elevated after five minutes of rest	Returning to baseline after five minutes of rest
**Neuromusculoskeletal measures (range of motion)**	High pain intensity High pain irritability Multiple motions reproduce pain	Low pain intensity Low pain irritability
**Palpation**	Hyperalgesia/allodynia	Normal response
**Pain pressure thresholds**	Low	High
**Conditioned pain modulation**	No change or decreases	Increases
**Temporal summation**	High	Low
**Starting exercise parameters** • Intensity • Sets x repetitions. • Frequency; Duration	**Aerobic^** • 40-70 % HR max or 8-13 rating of perceived exertion (RPE)- (RPE scale required if on beta blocker) • 2-5x/week • 20-60 minutes Resistance* • 40-70 % of maximum • 1-2 × 15-20 • 2-3x/week Isometric*^132^^,^^133^ • Up to 20 % of maximum • 2-5s for 10 minutes 2-5x/week	**Aerobic** • 55-90 % HR max or 11-16 rating of perceived exertion (RPE scale required if on beta blocker) • 4-7x/week • 30-60 minutes **Resistance** • 60-80 % of maximum • 1-3 × 8-12 • 2x/week Isometric*^134^ • Up to 80 % of maximum • Up to 5 × 45s • Up to 4x/week

^Consider shorter intervals to begin that add up to 20 minutes.

⁎ Consider starting with exercise at non-painful/least painful sites.^119^^,^^135^

Moderate intensity aerobic exercise has been a staple element of HTN management at all stages.^136^^,^^137^ High intensity aerobic exercise is safe and also appears to promote substantial decreases in BP in those with HTN, although there are limited high quality studies supporting this approach.^128^ Moreover, higher intensity training may not be tolerated in those with chronic pain, particularly early on during the rehabilitation process. It is important to recognize that, although exercise and lifestyle change are important management strategies in those with HTN, pharmacological intervention may be indicated in those with higher BP and/or those at higher risk for a cardiovascular event.^93^ Moderate to high intensity aerobic exercise has also been recommended for the best analgesic effect. However, Vaegter and Jones^21^ suggested that even very short-duration aerobic exercise can elicit EIH, which implies that intensity, or permutations of intensity and duration, may be more critical for achieving analgesia after aerobic exercise than either variable alone.

Depending on the patient’s pain and BP response to aerobic exercise/testing, resistance and/or isometric exercise may be a necessary and effective first step to address chronic pain and HTN. There is promising research emerging on the effects of isometric exercise and BP management.^136^^,^^138^ Large increases in BP can take place with resistance and isometric exercise**.**^121^ If there is a concern for large increases in BP, clinicians should consider using smaller musculature to begin**.**^138^

### Breathing/meditation

Emerging data suggest that meditation and/or breathing control exercises for 15-20 min/day can reduce BP in those with HTN ^39^ and improve ability in those with chronic musculoskeletal pain.^136^^,^^139^ Research on mindfulness meditation has demonstrated significant pain relief and greater activation in brain regions associated with cognitive modulation of pain, including the orbitofrontal, subgenual anterior cingulate, and anterior insular cortex.^140^

### Manual therapy

Manual therapy intervention has shown to increase PPT and facilitates impaired conditioned pain modulation in persons with lateral epicondylalgia of at least 6 weeks duration; however, the effects on BP were not reported.^141^^,^^142^ Yung et al found that non-thrust mobilization targeting the cervical spine in those with non-chronic neck pain results in acute changes in systolic BP.^143^ Other trials are underway investigating the effects of manual therapy on BP in those with HTN.^144^ While studies are limited, it seems plausible that improving pain may alter intertwined mechanisms involving chronic pain and HTN and reduce BP. Future studies are warranted.

## Future directions

We advocate for more research to identify and understand pathophysiological mechanisms and factors underlying the relationship between chronic pain and HTN. Understanding these mechanisms should lead to more targeted and succinct evidence-informed management for patients with chronic pain and HTN. Future research should also ascertain patients whose HTN is directly linked to chronic pain, and if targeting chronic pain can result in improved BP regulation. Finally, we recommend best practice framework and guideline development to assist clinicians in managing the patient with chronic pain and HTN.

## Conclusion

The personal and societal burden of chronic pain and HTN are significant. Although evidence suggests a relationship between chronic pain and HTN exists, there is much we have yet to learn. In conjunction with other health care providers, physical therapists can play a critical role in the management of patients with chronic pain and HTN. Identifying common risk factors, performing a thorough examination, and ongoing reassessment should allow progression of appropriate management that leads to successful outcomes for the patient with chronic pain and HTN.

## Declaration of competing interest

The author has no known competing financial interests or personal relationships that could have appeared to influence the work reported in this paper.

## Appendix Supplementary materials

Image, application 1

## Footnotes

Supplementary material associated with this article can be found, in the online version, at doi:10.1016/j.bjpt.2025.101229.
