# Supplementary material for: Rehabilitation management of the chronic pain-hypertension synergy: Proposal of an evidence-informed framework
Source: Braz J Phys Ther. 2025 Jun 3;29(5):101229. doi: 10.1016/j.bjpt.2025.101229 (PMC12167092; doi:10.1016/j.bjpt.2025.101229)
Supplement: Supplementary file 1 [file mmc1.pdf]

Rehabilitation Management of the Chronic Pain-Hypertension Synergy: Proposal of an Evidence-informed Framework

Supplementary material 1. Postulated model of acute blood pressure and pain responses in those who are generally pain-free versus individuals with chronic pain.[1]

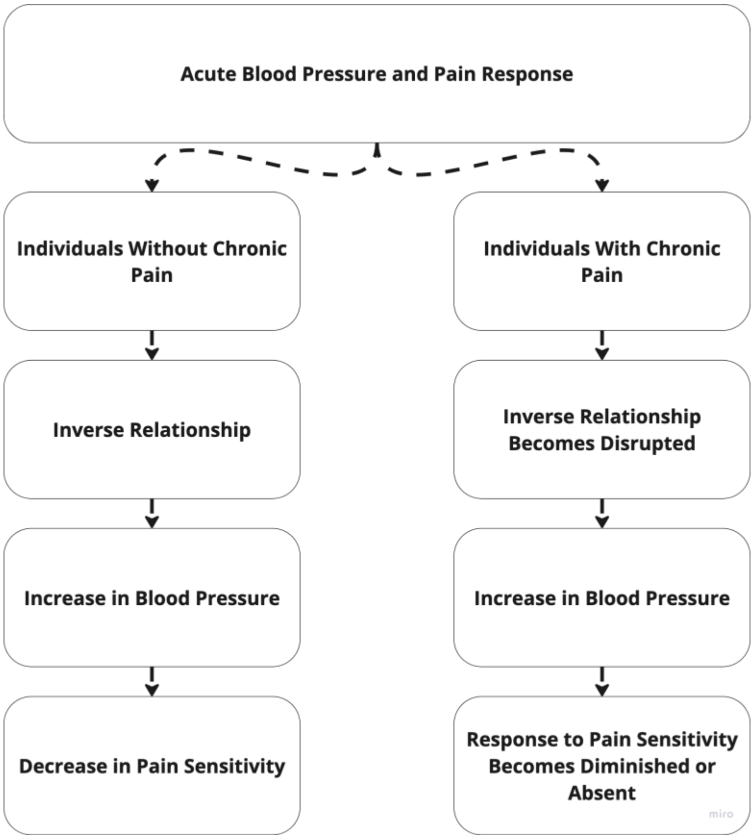

Supplementary material 2. Factors that influence alterations to the hypothalamus-pituitary-amygdala axis.[2,3]

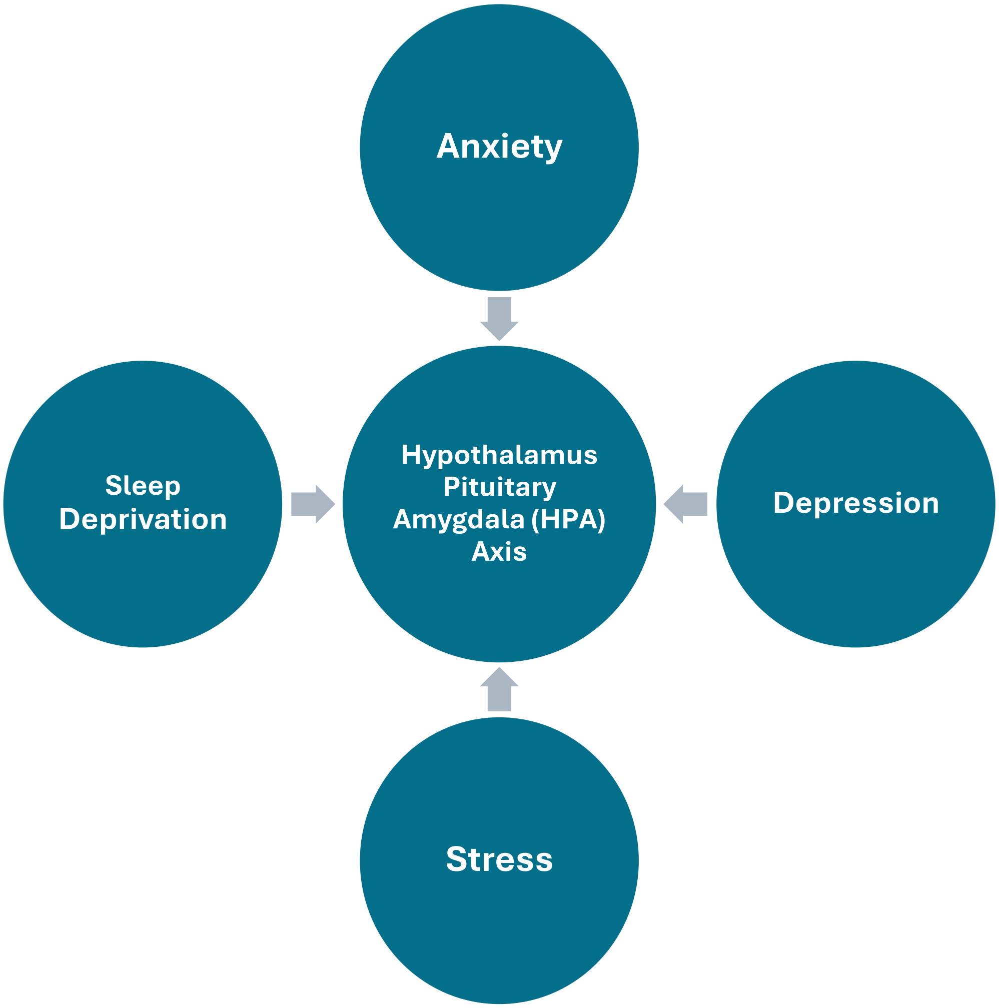

Supplementary material 3. Chronic blood pressure changes based on mode of exercise implementation. [4,5]

Exercise mode		Chronic changes
Aerobic training	Systolic	7.6 mmHg decrease
	Diastolic	4.7 mmHg decrease
	Systolic	5.7 mmHg decrease

Isotonic strength training	Diastolic	5.2 mmHg decrease
Isometric strength training	Systolic	4.3 mmHg decrease
	Diastolic	5.0 mmHg decrease

1. Bruehl S, Olsen RB, Tronstad C, et al. Chronic pain-related changes in cardiovascular regulation and impact on comorbid hypertension in a general population: the Tromsø study. *Pain*. 2018;159(1):119-127.
2. Kaufmann H, Norcliffe-Kaufmann L, Palma J-A. Baroreflex Dysfunction. *The New England journal of medicine*. 2020;382(2):163-178. doi:10.1056/NEJMra1509723
3. Zhuo M. Neural mechanisms underlying anxiety–chronic pain interactions. *Trends in neurosciences*. 2016;39(3):136-145.
4. Gkaliagkousi E, Gavriilaki E, Douma S. Effects of acute and chronic exercise in patients with essential hypertension: benefits and risks. *Am J Hypertens*. Apr 2015;28(4):429-39. doi:10.1093/ajh/hpu203
5. Edwards JJ, Deenmamode AH, Griffiths M, et al. Exercise training and resting blood pressure: a large-scale pairwise and network meta-analysis of randomised controlled trials. *British journal of sports medicine*. 2023;57(20):1317-1326.
